# Implementing a best-practice model of gestational diabetes mellitus care in dietetics: a qualitative study

**DOI:** 10.1186/s12913-019-3947-y

**Published:** 2019-02-14

**Authors:** Shelley A. Wilkinson, Maxine O’Brien, Sally McCray, Desley Harvey

**Affiliations:** 1 Department of Dietetics and Foodservices, Mater Health, Level 3 Salmon Building, Mater Health Services, Raymond Terrace, South Brisbane, Queensland 4101 Australia; 2 Mater Research Institute – University of Queensland, Mothers, Babies and Women’s Theme, Brisbane, Australia; 3 Alcohol and Other Drugs Services, Darling Downs Hospital and Health Service, Toowoomba, 4350 Australia; 4 Darling Downs Hospital and Health Service, Toowoomba, 4350 Australia; 5 Cairns and Hinterland Hospital and Health Service and College of Healthcare Sciences, James Cook University, Cairns, 4870 Australia

**Keywords:** Gestational diabetes mellitus, Health services research, Implementation, Medical nutrition therapy, Model of care, Qualitative

## Abstract

**Background:**

Translating research into clinical practice is challenging for health services. Emerging approaches in implementation science recognise the need for a theory–driven approach to identify and overcome barriers to guideline adherence. However, many clinicians do not have the capacity, confidence, or expertise to realise change in their local settings. Recently, two regional sites participated in a facilitated implementation project of an evidence-based model of gestational diabetes mellitus (GDM) care in dietetics, supported by a team at a metropolitan centre. This study describes (i) stakeholder experiences’, and (ii) learnings to inform implementation of the model of care (MOC) across Queensland.

**Methods:**

This qualitative descriptive study utilised semi-structured telephone interviews with staff involved in implementation of the MOC project at two regional sites. Eight participants were recruited; five participants were from one site. Interviews were transcribed and analysed to identify recurrent themes.

**Results:**

Four main themes were derived: (1) catalyst for positive change, (2) managing project logistics, (3) overcoming barriers, and (4) achieving change.

**Conclusions:**

A model of external facilitated implementation using an evidence-based decision making tool is an effective method of fostering health service change and is acceptable to staff. Key elements of the facilitation were building confidence and capacity in local implementers, through regular contact, encouraging local networking, linking to higher management support and assessing and/or influencing workplace or organizational culture. However, the balance between delivering clinical care while participating in a service change project proved challenging to many participants.

## Background

Gestational diabetes mellitus (GDM) is a condition increasing in prevalence with over of 5% of pregnancies affected [1]. Poorly controlled GDM can result in significant negative outcomes for mother and infant, including a significantly increased risk of type 2 diabetes mellitus, with associated patient and health system costs [2, 3]. Medical nutrition therapy (MNT) is the primary intervention for managing blood glucose levels in GDM and can result in significantly better blood glucose levels and less need for insulin, as demonstrated in validation of nutrition practice guidelines (NPG) in an American multi-centre trial [4]. These NPGs recommend women receive MNT according to an evidence-based appointment schedule with a dietitian [4] that incorporates a minimum of a one-hour individual initial counselling session and two review appointments [4].

Systematic delivery of MNT according to these guidelines does not occur in many Australian centres, with a wide variety of time allocations and models of care for delivering MNT to women with GDM [5, 6]. Known barriers include inadequate resourcing of dietitians in GDM clinics and unfamiliarity with NPGs [5–8] Further barriers include poor integration of dietitians into clinic procedures and poor awareness of the benefits of dietary intervention through regular dietetic contact for GDM management [5–8].

Implementation science methodologies recognise that translation of guidelines into practice requires a targeted, theory-driven approach to overcome barriers for service changes [9, 10]. This approach is being applied to develop and evaluate a statewide MNT model of care (MOC) by a lead site [8], local adaptation at two regional sites) [11], and a broader dissemination strategy. Briefly, following a barrier analysis [7] at the lead site, strategy selection using an evidence-based framework (i.e. the theoretical domains framework [10] and behaviour change wheel [12] was undertaken to overcome the identified barriers to guideline adherence. This implementation resulted in improved outcomes for women with GDM with significantly more women receiving best-practice care (less than 1% pre versus 51% post, *p* = 0.02) [8]. Additionally, fewer women required medication to manage GDM, improvements were reported in diet and physical activity measures, as well as in patient and staff satisfaction [8].

A decision tree tool was created through a combination and synthesis of the effective strategies from the lead site’s project to allow future sites to assess their own barriers and select evidence-based interventions to overcome them [8, 11]. The decision tree tool facilitated team decision making around reallocation, realigning and planning of resources, and allowed identification of and links to evidence-based nutrition training and resources in preparation for the implementation. Following site selection in a process outlined in Wilkinson et al. (2017) two regional sites were engaged in a hub (project team)-spoke (sites) model [11, 13]. Whilst the magnitude of the results varied between sites, the proportion of women seen according to best practice increased from 3.5 to 87.8% (*p* < 0.001) (Site 1) and nil to 4.8% (*p* = 0.09) (Site 2), and those on medication dropped by 3.4% (Site 1) and 9.1% (Site 2) [11].

This implementation approach at regional sites involved facilitators guiding sites to develop evidence-based solutions to local barriers. The external facilitators led local clinicians, managers and researchers, a “core” project team from each site, through phases of engagement, resource refinement, service mapping and monitoring, overcoming barriers and embedding the new MOC whilst monitoring clinical outcomes. While this stage of the project was successful in meeting its aims, barriers were faced during the implementation. The experiences of stakeholders directly affected by change can be beneficial in discerning what it takes to achieve successful implementation in specific contexts [14]. The aim of this paper is to describe the experience of stakeholders involved in implementation of best practice MNT and identify learnings to inform implementation at other sites [15].

## Methods

### Design and setting

This qualitative descriptive study utilised semi-structured interviews with staff involved in implementation of the GDM MOC in two regional sites. One site had ~ 2700 births annually and a GDM prevalence of 8% and the other site had~ 1200 births annually and a GDM prevalence of 9%. Sites were selected following an expression of interest distributed via a professional dietetic network across Queensland. Approval was obtained from institutional ethics committees (HREC/15/QCH/21–958 QA and HREC/15/QTDD/22-SA/QA).

### Participants

A purposive sample of practitioners were recruited from ‘core’ project members, including the GDM dietitian, self-nominated site project champion, dietetics project lead, plus key stakeholders (from nursing and medicine). The key stakeholders were able to provide variant perspectives on project implementation and were identified with the assistance of core team members. Eligible participants were invited to participate via email from a researcher. Those who responded received a written explanation of the study and provided written consent. Eight participants were recruited.

### Data collection and analysis

The Principal Researcher (SW) conducted all interviews by telephone. Interviews explored project experiences from commencement to completion, barriers and enablers to implementation, strategies to overcome challenges and recommendations for implementation at other sites. Interviews were 9–37 min duration. With consent, interviews were recorded on a digital recorder and transcribed verbatim for thematic analysis [16]. Two researchers (SW and DH) independently coded two interviews, agreed upon a coding framework and coded half the remaining transcripts each, noting illustrative text segments. SW and DH classified, sorted and synthesized codes in all transcripts to derive a smaller number of themes and sub-themes which were shared with other investigators and agreed by discussion and consensus. Finally, themes were examined with reference to the study aims and implementation science framework to identify learnings for other sites.

## Results

Four main themes were derived with reference to the study aims: [1] catalyst for positive change, [2] project logistics, [3] overcoming barriers, and [4] achieving change.

### Catalyst for positive change

Implementation of the project was described by participants as a catalyst for change in the local delivery of GDM services. Service improvement and patient benefits featured prominently in the participant responses as motivation for project participation, reflecting the expected outcomes for women in their care, data systems, and building a quality culture.

*What made us interested in it was basically that we didn’t know what was going on, we had no data because our data collection systems are basically non-existent and not accurate. (GDM5).*

Engagement with an external research team undertaking a systematic, statewide project also attracted clinicians to the project.

*I was keen to participate in it because it gave us exposure to doing a project with experienced researchers. I thought that there would be learnings and opportunities for observation and for growth …. (GDM4).*

### Managing project logistics

Participants balanced the known barriers with anticipation and enthusiasm for the project. Staffing and space were concerns as the project was introduced.

*I thought that it sounded like a good idea but I was a bit dubious as to whether or not we would be able to get funding for more staff and I think that was probably the main issue. (GDM1).*

Some participants expressed uncertainty about their project roles and the methods and processes. Despite initially feeling overwhelmed, the project structure was also reassuring, especially as the majority of the participants were project novices.

*Probably my only concern is that I’d never actually participated in a project before. So it was probably just with regards to the language used and getting my head around what was expected. (GDM6).*

The participants also talked of juggling clinical and project workloads, as they organised service changes, negotiated change, and undertook data entry for the project within their day-to-day practice.

*So I felt like it was quite hard to sustain and a bit stressful trying to fit and juggle it on top of a new workload.(GDM6).*

While some data entry issues were related to insufficient time or staffing, others reflect a lack of understanding of project methods and processes. This exacerbated and/or contributed to the burden of data entry for the clinicians in the project.

*… the initial data expectation was not as clear as … it should have been for someone with a non-project or research background. … While that was in the project outline somewhere, I think probably something like that I would have benefited from it being very, very clear.(GDM6).*

### Overcoming barriers

Major barriers were experienced at the sites, including resourcing constraints (space, time, and funding), and communication processes within sites. Participants reflected on how sites overcame barriers to both the model of care’s implementation and project delivery processes.

Some of the resourcing constraints to implementation had been anticipated by the project team and were highlighted in the decision making and problem solving tool provided to each implementation site by the lead site [11]. Others barriers emerged during implementation. These were space issues, funding, dietitian availability, and communication issues.

Space was a barrier mentioned by many participants at the beginning of the project.

*… it was a bit of a barrier for that first six months because there wasn’t always a room really available for a dietitian. We had to walk to another clinic room. (GDM2).*

Funding for increased access to dietitians to deliver the MOC was also an issue, with the prospect of withdrawal from the project at one site in the early stages.. Whilst many participants acknowledged the benefits of the data collected in providing insight into their processes of care and the resultant clinical outcomes, some participants felt the process of data management was laborious and suggested adjustments and/or improvements going forward.

*But data entry was a huge burden and is why we kind of didn’t continue using the database afterwards. (GDM5).*

Clear communication was also an issue for some participants.. This related to how the project was managed locally as well as within the delivery of the MOC.

Participants identified strategies that strengthened their approach to implementing the MOC including a strong focus on team communication and support and management buy-in.

*…. you have to involve everybody I think, as in anyone who’s going to be interested one way or the other, you just let everyone know. (GDM5).*

In addressing workload and system changes, participants reported the need for negotiation and refinement, resulting in local adaptation and acceptance.

*We tried to implement one of those [integrated antenatal clinician appointment] schedule at some point and the obstetric team just said no … but it didn’t mean that we couldn’t make our own schedule. So from a diabetes point of view we have our own schedule for dietetics (GDM1).*

Overcoming space and staffing barriers was achieved through negotiating for resources within the service (site A) and escalating resource issues to statewide allied health management (site B).

*the project …raised the profile of the importance of diabetes in pregnancy and so they were able to allocate people from the hospital over to us because they then saw it as a priority. So I guess within existing resources they were able to come up with more hours which was great. But it was a bit of a fight to get that. (GDM1).*

Support for this process of change resulted from engagement with and the support of a wide range of stakeholders, including management, medicine, administration staff, nursing, diabetes educators, and the other members of the project team.

*I think that was a key thing, having upper management support, that this is what we committed to so we need to do it. (GDM2).*

However, at times sites struggled with the level of changes required.

*However, even if we’ve got an extra admin person, which we did get because we had stakeholders that thought this was a great project, there was no space for that admin person. (GDM3).*

Those involved exhibited a persistent, problem solving approach, with a positive attitude.

*My process of work and my work ethic is that if you agree to do something you can’t then back out for no other reason than you haven’t been able to sort yourself out. I presented that fairly strongly.(GDM4).*

### Achieving change

Post project reflections capture the positive outcomes and improvements noted by all participants. System improvements included improved monitoring, follow up and review. Participants appreciated their achievements through the project.

*The [preparation] side of things was really difficult to organise but the project let us do it so now we’ve got it. But I don’t think without that kind of push that we could have got it done. (GDM5).*

Participants also acknowledged improved clinical practices within the broader model of care.

*I think that one-week review’s been really good, that we can do more individualised care and the post-natal reviews … So, it was really good to see that’s actually made some changes to the practice of what we do here. (GDM2).*

*She’s put in business case after business case over the last 20 years to get more help with dietetics and never really achieved it, whereas this project did achieve that (GDM1).*

Participants also noted that the changes adopted within the project improved patient outcomes,

*… the ladies who were participating in the project, a lot of them actually had a better outcome. (GDM7).*

Many participants appreciated the inclusion of dietitians in a stronger team approach.

*…*. *the systematic approach has helped … So it’s not just one lone dietitian saying, I need to see this patient. It’s everybody as a team is aware of what needs to be happening.(GDM1).*

Improvement in care aligned with increased dietitian confidence and in the dietetic service.

*We had all realised the value of dietetics a little bit more through the project because we’d had a quite positive experience from the project (GDM1).*

Participants reported a range of feelings on the project completion. These included relief, pride and greater regard for dietetics within the service.

*I felt like we had made some really important changes and that we had all realised the value of dietetics a little bit more through the project because we’d had a quite positive experience …(GDM1).*

Finally, some insights emphasized the need for improved support at the start of the project and how sites may better prepare for the process of service change. Participants talked of clarifying expectations and activities and ensuring sites have been fully informed.

*Probably a greater understanding of the project content prior to starting … would have been a good thing and that’s an individual thing. To understand the enormity of what you’re actually going to undertake and what the expectations are there. (GDM6).*

It was noted that preparation and orientation should allow for local differences and a focus on how local adaptation of the MOC can occur.

*So being told, well, in Brisbane we do this, is like one of the most insulting things you can say to a regional hospital. The fact that you’re given a guideline and saying, this is an example of what we do, but you do it to your own specifications and you alter it and tailor it to your own place. I think that’s what works best because then people make it their own and then it has more chance of succeeding, GDM1).*

Although each site faced different barriers, a common reflection was that sites should be prepared for the issues they faced.

*I would say to set clear guidelines of the time allocations and expectations, upfront (GDM 3).*

Communication and a preference for increased support from the research team was highlighted. Increased contact would enable information sharing and regular ongoing provision of support and supervision to sites.

*…more communication, site visits or even just phone calls or video conferences (GDM7).*

Participants also stressed the importance of regular communication with all stakeholders during site engagement.

*To ensure the communication process is smooth from beginning. Engage everyone, get all the stakeholders on board and keep everyone updated. I think this is quite important. (GDM7).*

Improvements to the data base system and refining data collection to local requirements was also recommended. The GDM Assist database system used, was considered more complex than necessary, thus increasing the time required for data entry.

*I think it’s just too clunky and I think that one page would have been perfect because you don’t have to flick through everything. (GDM2).*

Finally, participants discussed stakeholder engagement and resourcing, noting that in addition to broad stakeholder engagement, the composition of the team at the site was key to success.

*Having a small team of people - you need the right people. I think that’s what makes it work. Particularly for this project you need an endocrinologist, you need a diabetes educator, you need a dietitian and you need an obstetrician and a midwife, probably, as your team. I think if you’re missing support from your local team it won’t work. (GDM1).*

*I think making sure that the sites do involve the administration officers and the ones who are actually doing the work as well (GDM2).*

## Discussion

This study provides insights into the implementation of an evidence-based GDM MOC and suggests strategies for future dissemination. Our findings identified that staff optimism regarding potential for change within their service and resultant improved outcomes for women, and the project provided a catalyst for this change. However, the balance between delivering clinical care while participating in a service change project proved challenging to many participants. The MOC was implemented to different extents at each site; however participants at both sites reported that changes resulted in improved clinical practices and outcomes for women and an enhanced profile for dietitians in the service. Factors supporting change included engagement with an external project team, a robust project methodology, wide and ongoing site stakeholder engagement, multi-disciplinary higher-level management support, and a positive attitude.

The facilitated implementation approach using the hub-spoke model of site support and engagement mirrors the ‘hybrid-model’ suggested by Parker et al. in which opportunities of ‘local fit’ and ‘maximised buy-in’ from sites are balanced with tactical implementation decision and expertise from external facilitators [17]. Additional benefits were realised via engagement with senior management in the relevant hospital services and state allied health office. Rather than a prescriptive solution by the external facilitators, participants appreciated a guided process to overcome local barriers. This approach is desirable knowing that many clinicians work to implement research results by themselves, often in a largely non-formalized way across the organisation and not led by management [18]. However, greater local networking for problem solving could have been encouraged and a more formal local team and/or communication structure may benefit future project implementations [14]. Further, greater engagement and support from the external facilitation team, primarily around explicit expectation setting and regular, ongoing contact and communication are recommended [14].

Despite a promising approach, external facilitation has been poorly defined and rarely studied [19]. Few studies report its explicit use in implementation. Those that do vary in success. Our facilitated implementation experiences align with those who blended external and local stakeholder engagement, leading to increased uptake, adherence and improved quality, particularly when including local leadership engagement [20]. Those projects using facilitators trained externally, but operating locally within a health service have not been successful in integrating expected changes [21]. As in our study, Bidassie et al. noted that successful facilitated implementation is related to effective communication, relationship building, methods training, monitoring performance over time, and facilitating team-based problem solving [22].

This study is not without limitations. The limited number of interviews with clinicians outside the local project team (i.e. other key stakeholders of the service) may mean the experiences of this cohort may not translate to the larger GDM health service community and may have overlooked important processes or shortcomings. The findings may also reflect the specific local health service engagement of the implementation sites which were selected following an expression of interest and were therefore likely to be motivated to achieve change. Both sites were in regional areas and had a similar GDM prevalence. These findings may therefore not be applicable to less motivated sites with varied geographical and health service characteristics. Additionally, the interviewer was known to all participants which may have influenced responses.

## Conclusions

This study adds rich information to understand factors that support the translation of evidence into practice process. Findings suggest that a model of facilitated implementation using an evidence-based decision making tool is an effective method of fostering health service change. In addition to methodological expertise, key elements of the facilitation should include building confidence and capacity of local implementers through regular contact, encouraging local networking, linking to higher management support and assessing and influencing workplace or organisational culture.

## Abbreviations

GDM: Gestational diabetes mellitus
MNT: Medical nutrition therapy
MOC: Model of care
